# Integrated Glass Microfluidics with Electrochemical Nanogap Electrodes

**DOI:** 10.1021/acs.analchem.2c04257

**Published:** 2023-02-22

**Authors:** Sahana Sarkar, Ab F. Nieuwenhuis, Serge G. Lemay

**Affiliations:** Faculty of Science and Technology and MESA+ Institute for Nanotechnology, University of Twente, P.O. Box 217, 7500 AE Enschede, The Netherlands

## Abstract

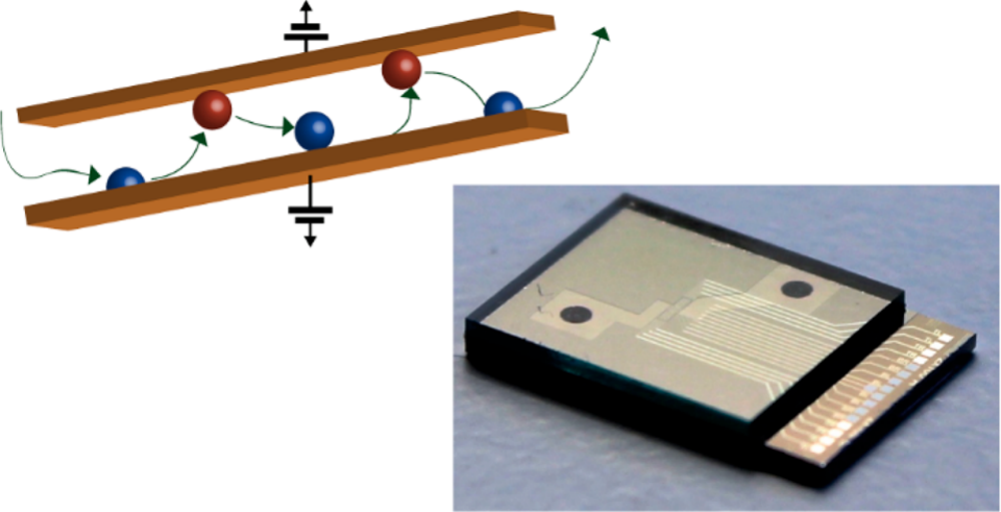

We present a framework for the fabrication of chip-based electrochemical nanogap sensors integrated with microfluidics. Instead of polydimethylsiloxane (PDMS), SU-8 aided adhesive bonding of silicon and glass wafers is used to implement parallel flow control. The fabrication process permits wafer-scale production with high throughput and reproducibility. Additionally, the monolithic structures allow simple electrical and fluidic connections, alleviating the need for specialized equipment. We demonstrate the utility of these flow-incorporated nanogap sensors by performing redox cycling measurements under laminar flow conditions.

Lab-on-a-chip platforms aim at creating plug-and-play systems that integrate the various functional elements (fluidics, sensing, electronics) to create sample-in–answer-out systems that are both accurate and portable.^1−9^ This drives the demand for downscaling methods so as to achieve faster and more sensitive assays.^4,6−9^ Electrochemical methods are well suited to this purpose as they require relatively simple components, yield electrical signals directly, and are readily miniaturized. Microfluidics, however, do not follow the same fabrication and scaling rules as electronics and sensing devices. The field has so far been dominated by soft lithography,^4,7,8,10^ with polydimethylsiloxane (PDMS) being the most widely used material. While useful for early prototyping, such methods can be difficult to integrate with other state-of-the-art fabrication techniques.^6−8,11^ Elastomers also typically have lower temperature resistance,^12,13^ can exhibit lower transparency,^14^ and can be incompatible with organic solvents.^13,15−17^ A common approach for wafer-scale fabrication is to create fluidic channels directly on a wafer using anisotropic etching, bulk micromachining, deep ion reactive etching (DRIE), and associated methods.^8,18−21^ The resulting structures are then bonded to wafers bearing electronics or sensors in order to create monolithic chip-based fluidic platforms. Such methods are not only favorable for large-scale production and commercialization, but also offer advantages such as greater mechanical strength combined with high-aspect structures.

We have previously reported nanogap electrodes for highly efficient redox cycling,^22^ as sketched in Figure 1a. Here, we report a new generation of devices incorporating wafer-scale integrated glass microfluidics (Figure 1b). No PDMS is involved, minimizing the risk of contamination and retention of analytes. The devices are rigid and can easily be interfaced to external tubing using conventional microfluidic interconnects.

**Figure 1:**
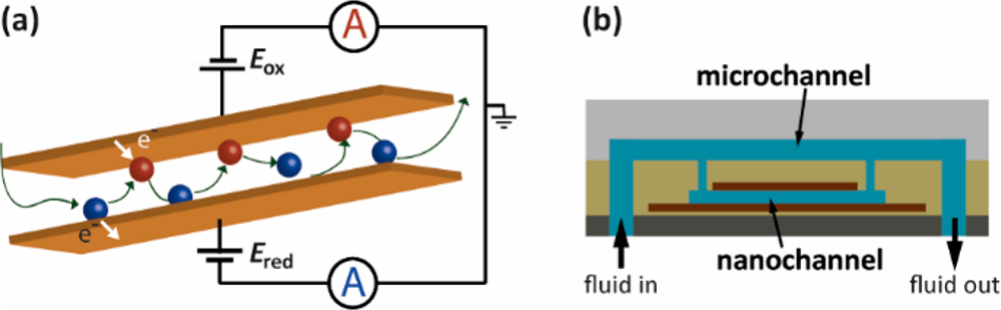
(a) Schematic diagram of redox cycling in a nanogap device. Chemically reversible redox molecules in the nanogap between two closely spaced plate electrodes undergo successive oxidation and reduction, thereby amplifying faradaic currents. (b) Schematic of parallel flow control using a SU-8 channel in parallel to the nanogap device for directed advection of the test solution.

## Materials and Methods

We employ parallel flow control, in which a microchannel is connected in parallel with the nanofluidic channel. Pressure at the inlet leads to flows in both micro- and nanochannels in a ratio given by the geometry of the channels, which allows controlling the velocity of the fluid in the nanogap device (Supporting Information).^23−25^

### Microfabrication

**Figure 2:**
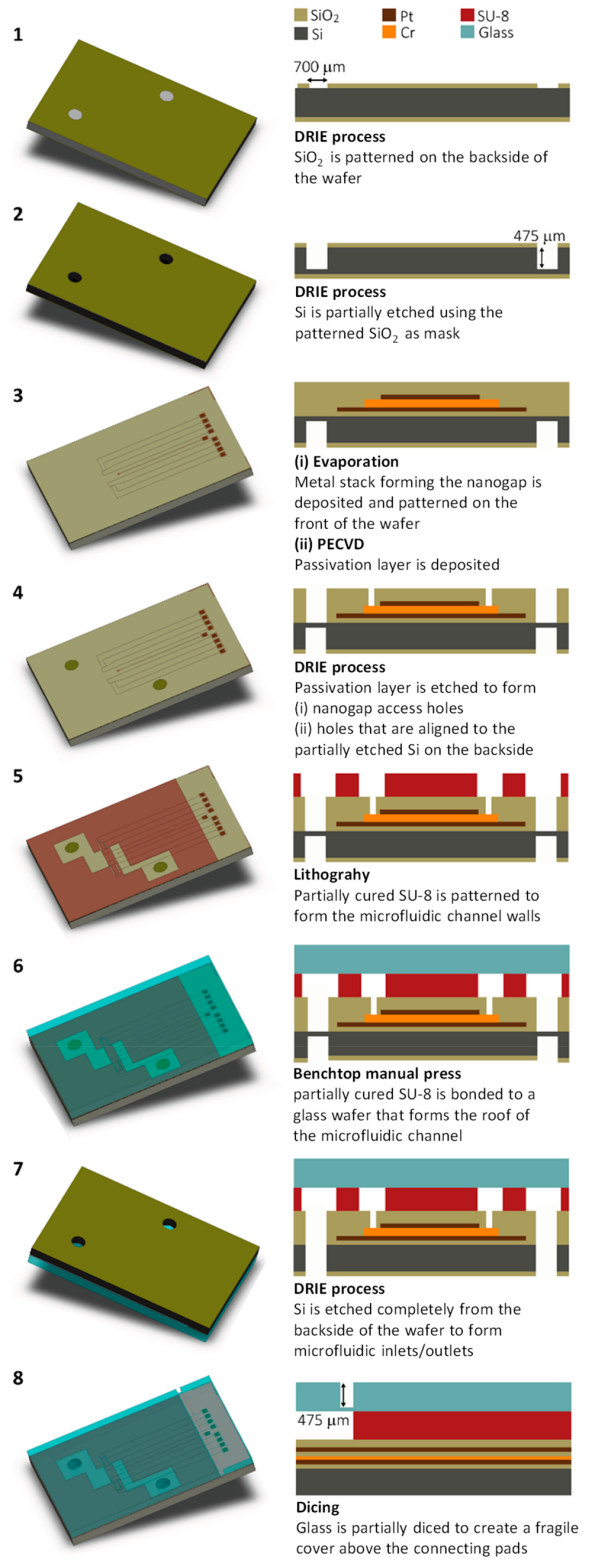
Schematic illustration (not drawn to scale) of the different stages of the fabrication process. The left and right columns show three-dimensional sketches and cross sections of a device, respectively.

Figure 2 shows our process flow for the fabrication of nanogap sensors incorporated with fluidic microchannels in a parallel flow configuration. A double-sided polished 4 in. Si wafer of a 525 μm thickness was used as the starting material. SiO_2_ of ∼500 nm thickness was thermally grown on both sides. In the first phase of fabrication, the back side of the wafer was processed to initiate the creation of the fluidic inlet and outlet. In step 1, a positive photoresist (OIR 907-12, Arch chemicals) of thickness 1.7 μm was deposited and patterned by photolithography and then hard baked (100 °C) for 10 min. This served as the mask for etching the oxide layer by DRIE using an Adixen SE instrument (Alcatel model AMS SE). Two markers, placed on the wafer edge diametrically opposite to each other, were also etched in the oxide layer during this step. In step 2, the Si was etched down to a depth of ∼475 μm, the previously patterned silicon oxide effectively acting as a mask. A DRIE (Bosch) process was chosen once again due to its directionality such that straight edges could be achieved. This step was performed on an Adixen DE instrument (Alcatel model AMS DE). This left ∼50 μm of material intact so as not to interfere with spin coating resist on the other side of the wafer. Thereafter, in step 3, the wafer was flipped over, and nanogap devices were fabricated on the front side following a previously reported process.^26^ In short, a 20 nm thick Pt bottom electrode, a 60 nm thick Cr sacrificial layer, and a 120 nm thick Pt top electrode were sequentially deposited by electron-beam evaporation and patterned using a lift-off process based on a positive photoresist (OIR 907-17, Arch chemicals). The wafer was carefully aligned to the markers on the backside of the wafer in order to ensure the alignment of the micro- and nanofluidic inlet/outlets. Afterward, a passivation layer of silicon dioxide (SiO_2_) and nonstoichiometric silicon nitride (SiN) consisting of 120 nm/360 nm/120 nm SiO_2_/SiN/SiO_2_ was deposited using plasma-enhanced chemical vapor deposition (PECVD). In step 4, the passivation layer was patterned and etched in two different regions: first to form the inlet and outlet of the nanochannels by exposing the sacrificial chromium layer from the top and second to provide an opening to the fluidic inlet/outlet etched earlier from the back side of the wafer. In step 5, SU-8 2005 was spin coated on the top of the nanogap structures. With a thickness of ∼6 μm, the resist was soft baked by ramping up the temperature to 95 °C over 30 min to avoid SU-8 cracking followed by cooling down to room temperature. Following exposure to UV light, the resist was developed; the patterned structures created the walls of the SU-8 microchannels, defining their height. The conventional postbake step was intentionally not performed, as a result of which the SU-8 only partially cured. Subsequently, this patterned Si wafer was irreversibly bonded^27^ to a 4 in. Borofloat glass wafer (525 μm) at an elevated temperature and pressure using a hydraulic press (180 °C, 1.4 MPa) for 1 h (step 6). This step was done within a few hours of patterning the SU-8 while it was still soft. Finally, in step 7, the leftover silicon blocking the inlet/outlet channels was etched away from the backside using the same DRIE process as in step 2 but with a different etcher (SPTS model Pegasus) that allows a stack of wafers (Si/SU-8/glass) to be processed. Additionally, the electrical connection pads needed to be exposed for electrical connections. To achieve this, the SU-8 was originally patterned in such a way that it did not cover the parts of the wafer that held the electrical contact pads. It was thus possible to cut through the glass wafer partially (down to 475 μm) around the connection pads without disturbing the remaining stack (step 8). Finally, the entire wafer was diced into individual chips. The final chip included a fragile but protective glass part extending over the connection pads that was broken off with tweezers immediately before using a chip.

We chose this approach over commonly used methods such as anodic bonding because the latter require the application of high temperatures and voltages. This is incompatible with our nanogap sensors since Cr and Pt interdiffuse at elevated temperatures.

### Fluidic and electronic interface

**Figure 3:**
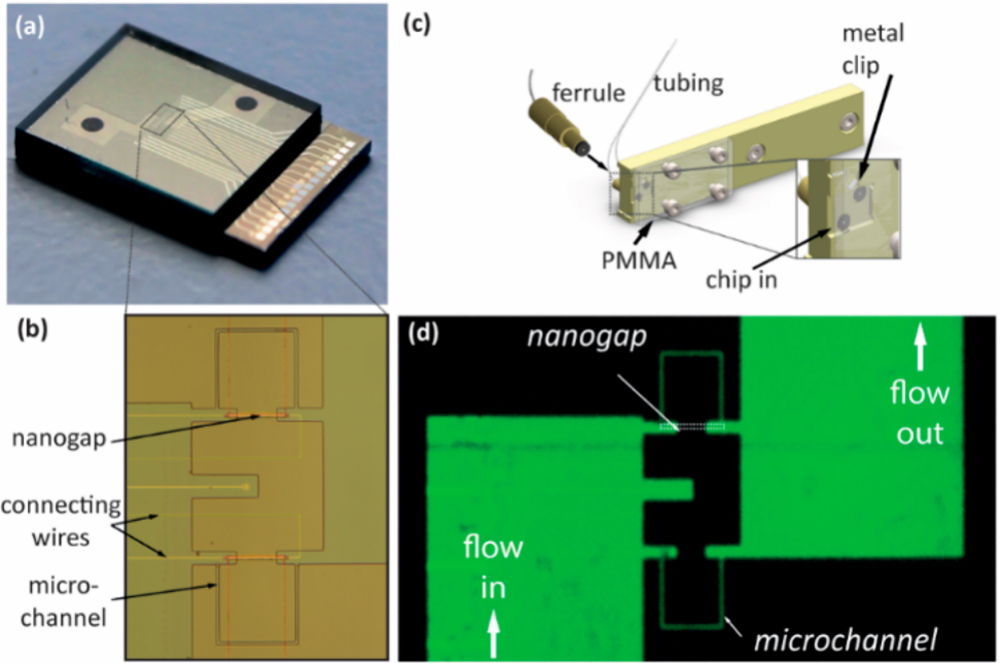
(a) Photograph of the microfluidic integrated nanogap sensor. (b) Optical micrograph of the active region of the device. (c) Assembly of the fluidic device for measurements. Fluid is introduced via two threaded holes in the backside of the holder. (d) Optical image of the channels filled with a fluorescent dye (fluorescein) at an injection rate of 350 nL/min.

A completed device is shown in Figure 3a and b. Microfluidic connections were made via the 700 μm holes on the backside of the chip. To do so, the chip was mounted on a PEEK substrate holder as shown in Figure 3c and held in place by a poly(methyl methacrylate) (PMMA) plate. Gaskets were then pressed against the backside of the chip for leak-proof connections by screwing microfluidic ferrules into the holder (Idex microfluidics for nanoport assemblies, catalogue no. F-123 H and N 123-03, respectively). Due to wear down of the blades during the process of dicing, the lateral dimensions of the chip had an error of up to ∼50 μm. To accommodate these variations, the diameters of the holes in the chip were made about twice the size of the opening of the gasket (360 μm diameter). A metallic clip was used as a spring to reproducibly align the holder and the holes in the chip.

The inlet tube was connected to a conventional syringe pump (Pump 11 Pico Plus Elite, Harvard Apparatus). Based on the geometry of the devices, the average fluid velocity over the cross section of the nanochannel was 1.4 mm/s per μL/min imposed by the pump (Supporting Information).

Prior to the measurements, chromium etchant (BASF, Chromium Etch Selectipur) was circulated through the inlets in order to etch the sacrificial layer and release the nanochannel. The electrodes were cleaned by cycling voltammetry in 500 mM H_2_SO_4_.

## Results and Discussion

To test the quality of the wafer adhesion, saturated solutions of fluorescein sodium salt (Sigma-Aldrich; catalogue no. F6377) were passed through devices at an applied inlet pressure of 2.5 bar for ca. 2 h and monitored using light with a wavelength of 460 nm. Figure 3d shows an optical image of a device at the conclusion of such a test. No fluorescence was observed outside the channel areas in these tests, indicating that the SU-8 walls provide a good seal with the top glass wafer. On the other hand, imposing flow rates higher than 5 μL/min (pressure 20 bar) typically caused the glass layer to delaminate from the SU-8 layer (Figure 2, step 6) or the external microfluidic components to fail.

Reversible adsorption of analyte molecules and its irreversible counterpart, electrode fouling, are commonly observed during electrochemical analysis, particularly with biological samples. This affects the overall sensitivity, detection limit, reproducibility, and response time of electrochemical sensors.^26^ Adsorption, whether reversible or irreversible, is of particular concern in micro/nanofluidic devices due to their inherently high surface-to-volume ratio and the inability to polish the electrodes.

As a first test of our microfluidic-enabled devices, we performed cyclic-voltammetry measurements using ferrocene dimethanol (Fc(MeOH)_2_, Sigma-Aldrich, catalogue no. 372625) as redox species since its response is well characterized for nanogap devices.^26^ Nanogaps of dimensions 100 μm × 3 μm × 65 ± 5 nm were used for the experiments. The shape of the voltammogram is known to be influenced by reversible adsorption, the main manifestation being a hysteretic response even at relatively slow scan rates.^26^

**Figure 4:**
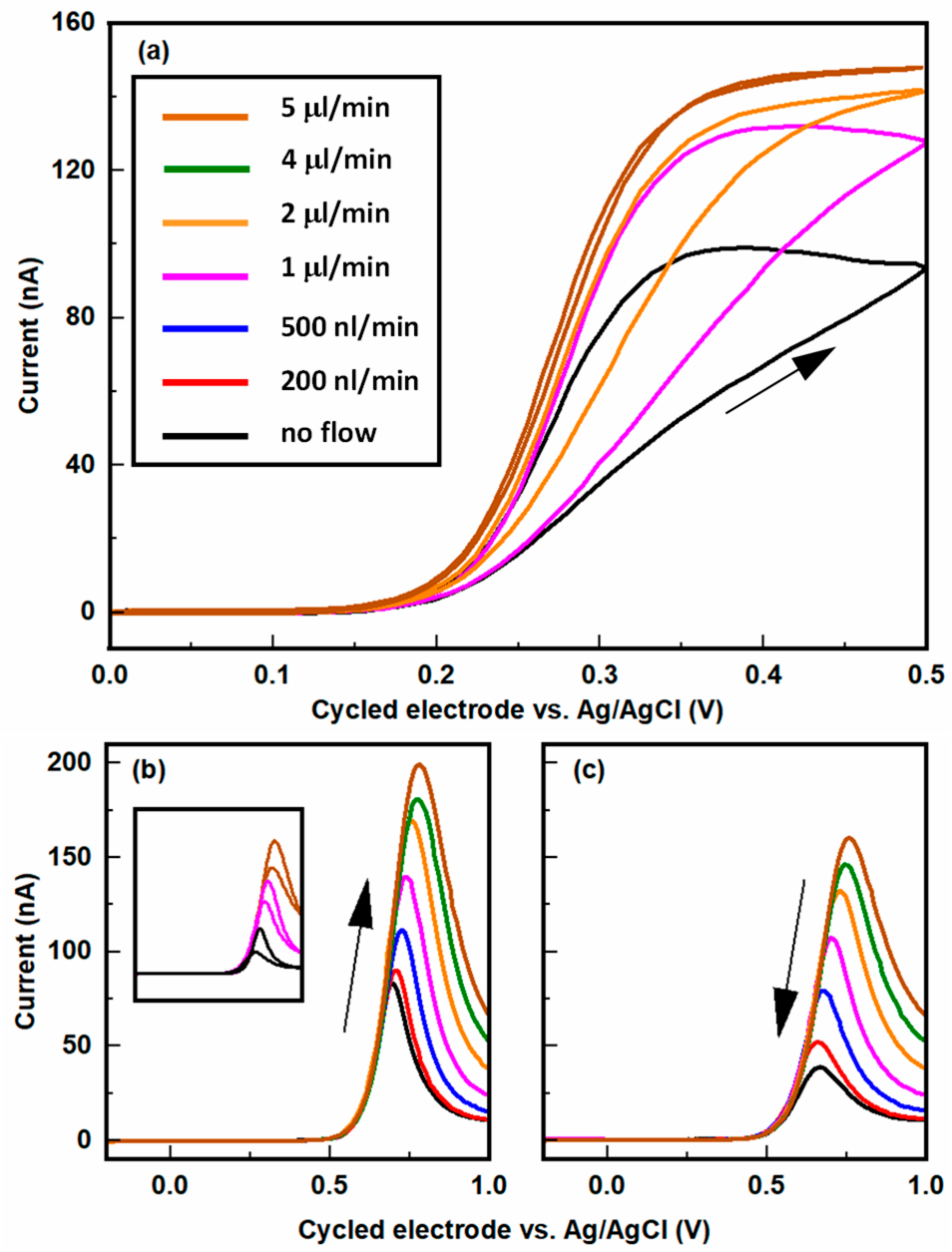
(a) Cyclic voltammograms at different pump flow rates during redox cycling (sweep rate 50 mV/s) with 1 mM Fc(MeOH)_2_ in a 0.1 M KCl aqueous solution. The potential of the top electrode was cycled, and the bottom electrode was maintained at 0 V with respect to Ag/AgCl. Only the oxidizing (top) electrode current is shown; the reducing current is essentially equal in magnitude. The hysteresis caused by reversible adsorption of Fe(MeOH)_2_ on the Pt electrodes decreased with increasing flow rate and essentially disappeared at high flow rates. (b, c) Same as (a) for 210 μM dopamine in a 10 mM PBS solution at different flow rates. The forward (b) and backward (c) scans are shown separately for clarity. The same data are shown as complete cycles in the inset for three selected flow rates to better illustrate the marked hysteresis.

Figure 4a shows cyclic voltammograms obtained at various flow rates for 1 mM Fc(MeOH)_2_ in a 0.1 M KCl aqueous solution. The potential of the top electrode was swept while that of the bottom electrode remained constant at 0 V. As previously reported, for such long devices, the voltammograms can exhibit pronounced hysteresis or low flow rates due to reversible adsorption of the redox species.^26^ The magnitude of this hysteresis decreases with increasing flow rate, however, and essentially disappears at a fluid injection rate of 5 μL/min (average fluid velocity 7 mm/s), reflecting the fast replenishment of the solution in the nanochannel and the corresponding maintenance of near-steady-state conditions in the nanogap device. This explicitly demonstrates how advective flow enhances the time response of redox-cycling-based nanofluidic detectors.

Dopamine is a notable neurotransmitter implicated in brain functions that include pleasure reward, memory, and behavior. While several electrochemical methods of detection have been developed, for example, fast-scan voltammetry at carbon electrodes,^28^ accurate determination of dopamine concentration still represents a challenge. One reason is interference from compounds such as ascorbic acid that are present in large excess. Another is that dopamine is a notorious fouling agent, forming highly reactive intermediates that result in free radical polymerization and ultimately in the formation of melanin. This precipitates unto the electrode surface and creates a blocking layer, inhibiting further surface-dependent redox chemistry unless this is mitigated via the choice of electrode material or the introduction of surfactants.^29,30^ Dopamine is a chemically reversible electroactive species, and therefore, nanogap sensors provide a high level of signal amplification via redox cycling.^31^ It has previously been shown for catechols that this can help mitigate interference from ascorbic acid.^32^

To test the behavior of dopamine under advective flow in a nanochannel, dopamine hydrochloride (SigmaAldrich, catalogue no. H8502) was prepared with a concentration of 210 μM in phosphate-buffered saline with a pH of 7.4 (SigmaAldrich, catalogue no. P4417) as a supporting electrolyte. Cyclic voltammograms were recorded at different flow velocities. The bottom electrode was kept at −200 mV vs Ag/AgCl while the top electrode was kept between −200 mV and +1000 mV. Figure 4b and c shows the redox cycling current for different flow rates. In each case, the voltammograms exhibited a sharp maximum followed by a gradual decay at large overpotentials. The negative sweeps exhibited hysteresis in the form of a lower peak current, as reported for catechol.^32^ The magnitude of the maximum current also increased monotonically with increasing flow rate. The response of dopamine in redox cycling is complex as it depends on analyte adsorption, electrode fouling, and local changes in pH due to proton gradients being created during redox cycling.

## Conclusions and Outlook

We introduced a process flow based on DRIE and SU-8 wafer bonding that allows fabricating glass-based nanofluidic devices with embedded nanogap electrodes suitable for highly efficient redox cycling. In addition to avoiding the use of PDMS, this approach delivers robust monolithic devices that facilitate both fluidic and electronic interconnections. The approach is not limited to nanogap electrochemical transducers and can be easily generalized to other electrochemical and fluidic structures such as parallel-flow-control mixers.^33^ We anticipate that such devices can increase the access to electrochemical nanofluidic devices for a broader base of users and analytical applications.

## Supporting Information Available

Explanation of parallel flow control (PDF)
