# Supplementary material for: Integrated Glass Microfluidics with Electrochemical Nanogap Electrodes
Source: Anal Chem. 2023 Feb 22;95(9):4266–70. doi: 10.1021/acs.analchem.2c04257 (PMC9996602; doi:10.1021/acs.analchem.2c04257)
Supplement: Supplementary file 1 — ac2c04257_si_001.pdf [file ac2c04257_si_001.pdf]

Supporting Information

Integrated glass microfluidics with electrochemical nanogap electrodes

Sahana Sarkar, Ab F. Nieuwenhuis and Serge G. Lemay
MESA+ Institute for Nanotechnology and Faculty of Science and Technology, University of Twente, PO Box 217, 7500 AE Enschede, the Netherlands

Table of contents

Parallel flow control 2
References 3

Parallel flow control

Fluid entering the device encounters two parallel channels, the SU-8 microchannel and the nanogap, and is split between these two pathways as shown in Figure S1. For a Poiseuille flow in this configuration, the flow rates in the two channels is given by the ratio of their hydraulic resistances, R, such that[1, 2]

$$\frac{Q_\mu}{Q_n} = \frac{R_n}{R_\mu} \approx \frac{\left(1-\frac{0.63h_\mu}{w_\mu}\right)}{\left(1-\frac{0.63h_n}{w_n}\right)} \frac{h_\mu^3 w_\mu L_n}{h_n^3 w_n L_\mu} \ . \tag{S1}$$

Here w, h, and L represent the width, height and length of the channels, respectively. The dimensions of the nanochannel are defined by the volume initially occupied by the sacrificial Cr layer. For a nanogap of length 100 µm and cross sectional area of 5 µm × 65 nm, the resistance has a value $R_n \approx 9 \times 10^{20}$ Pa s/m^3. For a microchannel that is 1084 µm long and with a 6 µm × 5 µm, a flow rate ratio of 24,000:1 is expected between the micro- and nanochannels. In practice, two devices and two parallel fluidic channels were present on a single chip. The flow rate through each nanochannel was thus reduced by a factor of approximately 48,000 compared to the flow rate provided by an external syringe pump.

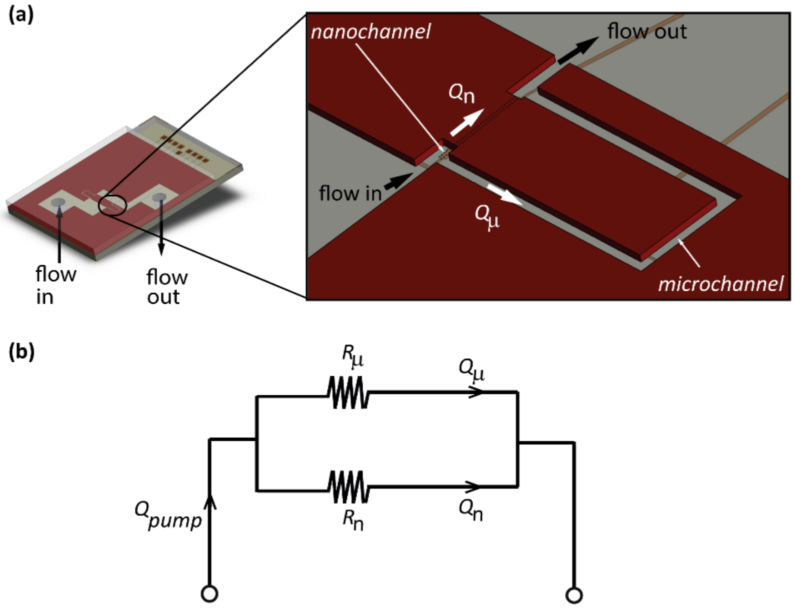

Figure S1. (a) Schematic of the fluid flow in the chip. (b) Equivalent circuit for the fluidic configuration where R_n and R_μ represent the channel resistances while Q_n and Q_μ are the flow rates in each channel.

References

1. Bruus, H., Acoustofluidics 1: Governing equations in microfluidics. *Lab Chip* **2011,** *11* (22), 3742-3751.

2. Mathwig, K.; Mampallil, D.; Kang, S.; Lemay, S. G., Electrical Cross-Correlation Spectroscopy: Measuring Picoliter-per-Minute Flows in Nanochannels. *Phys. Rev. Lett.* **2012,** *109* (11), 118302.
